# Impact of Anxious and/or Depressive Reactive State on the Effectiveness of Rehabilitation of Patients with Multiple Sclerosis

**DOI:** 10.3390/medicina60060941

**Published:** 2024-06-04

**Authors:** Tatjana Bućma, Igor Sladojević, Lena Topić Arambašić, Natalija Jeremić, Bosa Tomić

**Affiliations:** 1 Institute for Physical Medicine, Rehabilitation and Orthopedic Surgery “Dr Miroslav Zotovic”, 78000 Banja Luka, Bosnia and Herzegovina; lena.topic@yahoo.com (L.T.A.); curicnatalija@yahoo.com (N.J.); bosatomic@gmail.com (B.T.); 2 University of Banjaluka, Faculty of Medicine, 78000 Banja Luka, Bosnia and Herzegovina; igor.sladojevic@med.unibl.org

**Keywords:** multiple sclerosis, quality of life, psychological reactive states, rehabilitation, functional status

## Abstract

*Background and Objectives*: Rehabilitation is a part of the comprehensive treatment of multiple sclerosis (MS). If present, psychological reactive states limit the results of the rehabilitation. The objectives were to determine the impact of psychological reactive states in these patients on the functionality obtained by rehabilitation and QoL, and to determine the connection between the objective and subjective evaluation. *Materials and Methods*: Based on the Hospital anxiety and depression scale, the patients were divided into a group with anxious and/or depressive reactive state and a group without the reactive state. The values of functional scores—the Berg Balance Scale (BBS) and the Expanded Disability Status Scale (EDSS), as well as the parameters of the QoL-Physical health Component Score (PCS) and the Mental health Component Score (MCS)—were determined at the beginning and at the end of the rehabilitation. *Results*: There was a statistically significant difference between the BBS, EDSS, PCS, and MCS groups at the beginning and the end of the rehabilitation in both groups. A statistically significant difference at the beginning and the end of the rehabilitation between the groups was found only in PCS and MCS. A highly statistically significant correlation between EDSS and PCS, and EDSS and MCS, was found only in the group without the reactive state. *Conclusions*: Although rehabilitation leads to an objective improvement of functionality in patients with MS, the presence of the anxious and/or depressive reactive state limits the results of rehabilitation and leads to discrepancies in the aforementioned objective assessment and the patient’s subjective experience through the evaluation of their QoL.

## 1. Introduction

Multiple sclerosis (MS) is the most widespread chronic inflammatory disease of the central nervous system (CNS) and affects more than two million people worldwide. The most common symptoms are motor weakness, sensory disorders, gait and coordination disorders, and optic neuritis [1]. Besides these, patients may have bowel and bladder disturbances, sexual dysfunction, pain, speech and swallowing disorders, cognitive dysfunction, and mental and psychological disorders that significantly limit the abilities in everyday life and social activities of the patient [2]. 

Rehabilitation is increasingly recognized as an essential part of the comprehensive treatment of patients with MS by improving the effects of treatment options [3]. Studies [4,5] showed that patients tolerated the programmed exercises well. They were a safe and effective ways to improve the quality of life (QoL) of these patients by optimizing everyday functioning and increasing their participation in different spheres of life. An important part of the treatment of patients belongs to methods such as proprioceptive neuromuscular facilitation and Bobath neurodevelopmental treatment. Modern technology, which is used in neurorehabilitation and in the treatment of patients with MS, such as robotically assisted rehabilitation, provide significant advantages in the effects of treatment of these patients [6].

The literature indicates an increased prevalence of depression and anxiety in patients with MS compared to the general population [7]. The prevalence of anxiety disorder in patients with MS is 22.1% on average, while a high level of anxiety has been found when using a self-report questionnaire [8]. The prevalence of depression ranges from 4.98 to 58.9%, with an average of 23.7% [9], while it was 30.5% in a paper by Boeschoten [8]. Appropriate confrontation with the disease is important in these patients, as is the use of mature defense mechanisms, social support, and lower levels of stress as protective factors [10].

The literature data indicate that the patients with MS have a lower QoL than the general population [11,12]. Torp Løkkeberg et al., in 2022 [13], have described that people have a QoL when they can cover elementary biological needs, have human contacts, purposeful jobs, and have diverse, exciting, and interesting experiences in life. QoL is affected by numerous factors that are connected to the disease, such as the degree of disability or the type of MS, as well as other factors such as social support, education, age, or employment [14]. It is considered [15] that the lack of social support is connected with depression, anxiety, and negative QoL. The QoL of these patients is also affected by general well-being, psychological function, and social functions that are not directly connected to the neurological disease but are considered by the patients to be even more important determinants of their health condition than the impaired physical function is [16]. 

Measurements of health-related QoL as a relevant assessment of the progression of the disease, treatment response, and level of assistance needed by the patients [17], have been considered in the last decades. Therefore, researchers suggest that the test score of disease-related QoL be included along with the other parameters, clinical and biological, when assessing the response to the treatment of the disease [18].

According to the mentioned factors and the experience through practice, the goal of the research was to determine the impact of psychological reactive states in patients with MS on the functionality obtained through the process of complex rehabilitation, as well as on the patient’s QoL. The hypothesis is that there is no connection between the objective assessment of the patient’s functional state and his/her subjective assessment of the physical and mental state.

## 2. Materials and Methods

The research was carried out as a prospective interventional study at the Institute for Physical Medicine, Rehabilitation, and Orthopedic Surgery “Dr. Miroslav Zotović” Banja Luka, Bosnia and Herzegovina, from March 2022 to November 2023. In the research period, 74 people of both genders with the diagnosis of MS were treated at the Department for Neurorehabilitation IIA. 

The inclusion criteria were as follows: Confirmed diagnosis of MS, patients between 18 and 70 years of age, no disease relapse in the past month, no confirmed psychiatric diagnosis, the cognitive function according to Mini Mental examination (MMSE) was assessed at ≥24, and no associated disease, whether neurological, rheumatological, or orthopedic, which would lead to functional limitation, and no significant damage to the function of the subject’s cardiovascular and respiratory system. 

The exclusion criteria were as follows: Deterioration of the underlying disease during the research, newly confirmed neurological, rheumatological, or orthopedic disease, worsening of cardiovascular and respiratory system function, and development of some inflammatory disease with febrility (urinary system, respiratory system, etc.).

Participants in the study were selected by a specialist of physical medicine and rehabilitation who was the leader of the rehabilitation team. Patients who satisfied the inclusion criteria were presented with the information for patients, and if they accepted to participate in the study, they had to sign the informed consent, approved by the Institutional Ethics Committee. 

Measurements were made after the examination by the specialist in physical medicine and rehabilitation and the determination of the general and functional status. The patients were tested by a psychologist using the Hospital Anxiety and Depression Scale (HAD), which was shown by the studies to be suitable for the identification of mood changes in persons with MS with good metric properties [10], high specificity, and sensitivity [19]. HAD has been presented as a reliable instrument for screening clinically significant anxiety and depression in patients attending a general medical clinic. This scale has also been shown to be a valid measure of the severity of these disorders of mood and, therefore, the repeated administration of the scale on subsequent visits to the clinic will give useful information [20] to the physician. 

Based on HAD, the patients were divided into two groups—group I, into which patients who had HAD results of >7 were classified, which means that they probably or certainly had anxiety and depression symptoms; and group II, where the HAD values were ≤7, indicating that the patients did not have any anxiety or depression symptoms. There were 24 patients classified into group I, and 34 patients into group II. 

Before the start of the rehabilitation process, graduated physiotherapists assessed the functional status of all patients using the Expanded Disability Status Scale (EDSS), and Berg Balance Scale (BBS), which were also used to track the progress during the rehabilitation. EDSS is considered suitable for discovering effectiveness in clinical interventions and monitoring the progression of MS. It is the most widely used tool for measuring the outcome of the disease that is internationally accepted [21,22]. It has a range from 0, defined as “neurologically normal findings”, to 10, representing the fatal consequences of MS. BBS is valid and reliable, and is most often used for assessment of the balance and risk of falling in patients with MS. It also assesses the static balance of sitting, posture change, transfers, and balance in standing position. BBS ranges from 0 to 56; higher scores mean better balance [23,24]. 

The last test was the assessment of the QoL of patients with a standardized SF36 test. It is a widely validated and used tool for measuring QoL in MS that uses eight subscales that can be summarized into two results, a physical health component score (PSC) and a mental health component score (MSC) [25]. Higher scores indicate better QoL. 

The interventional protocol was as follows: Rehabilitation lasted for five weeks, with physical therapy five days a week that was adjusted to the individual needs of patients. The first segment was kinesitherapy, which included a program lasting 45 to 60 min, in accordance with the patient’s abilities. The central part of the treatment was the Bobath concept, which is a valid and recognized concept with positive impacts on balance, postural control, and indirectly, on the adaptive index of neuroplasticity [26]. The concept emphasizes the critical role of postural stability [27], which is necessary for selective movements and balance. It is a good tool that has been a part of the comprehensive treatment of patients with MS [28,29]. Walking, balance, and coordination exercises were also conducted, as were exercises for muscle strength improvement, range of motion (ROM) exercises, breathing exercises, and walking exercises between parallel bars. Afterward, pelvic floor strengthening exercises were conducted, depending on the type and existence of damage to the sphincter’s function (whether due to damage of CNS or insufficiency of detrusor of the bladder). Before kinesitherapy, the Novafon^®^ device (Novafon, Weinstadt, Germany) was used in patients with spasticity. The device causes a physiological effect by vibration massage, where gentle vibrations from the surface of the skin transfer to muscles and cause relaxation, which visibly stabilizes the tonus and enables the implementation of the program. 

The second interventional strategy was occupational therapy, with a duration of 30 to 45 min. Also, the activities of muscle strengthening, ROM exercises, proprioceptive training, and sensory reeducation were used. Robotically assisted training was also conducted on a medical device Armeo^®^Senso (Hocoma, Volketswil, Switzerland) for the upper extremities. The device uses self-initiated, active, motivating, and repetitive arm movement in a 3D workspace for patients with mild to moderate impairments. It uses three sensors and a manual module for monitoring the movement and providing feedback information on improved performances in real-time. All patients were capable of performing this training. The goal of the work on this system was to increase the active range of movement, increase strength and endurance, facilitate self-initiative walking, improve coordination, improve grabbing function, increase selective control, reduce spatial neglect, and improve mobilization of the patient. At the end of the rehabilitation, EDSS, BBS, and SF36 testings were repeated. All patients were evaluated by the same examiner. 

### Statistical Analysis

The obtained results were statistically processed with SPSS version 29 software (IBM corporation, New York, NY, USA), using methods of descriptive statistics, Pearson’s correlation coefficient, and *t*-test. A *p*-value of <0.05 was considered statistically significant.

## 3. Results

Inclusion criteria were met by 58 male/female patients. Sixteen patients had to be excluded from the study (two were out of age range, two had hip arthrosis, one had a lower limb injury, one had diabetic polyneuropathy, and four had urinary tract infections; one patient had an MS relapse, three had unstable angina pectoris; one patient had chronic obstructive pulmonary disease, and one patient had an MMSE score of 22). 

The duration of the disease was statistically significantly longer in patients in group II. Also, EDSS and PCS values were higher in group II, both before and after the therapy. There was no statistically significant difference between the life age of patients in one versus the other group (Table 1). 

There was a highly statistically significant difference in BBS, EDSS, PCS, and MCS between the beginning and end of rehabilitation in both groups (Table 2). 

Comparing the values between the groups before and after the rehabilitation, a highly statistically significant difference was found between PCS and MCS before, and MCS after the rehabilitation, as well as a statistically significant difference in PCS after the rehabilitation. There was no statistically significant difference in the parameters of EDSS and BBS between the groups before and after the rehabilitation (Table 3).

There was a highly statistically significant correlation between EDSS and PCS, i.e., between the EDSS and the MCS values in the group without the anxious and/or depressive reactive state, while there was no statistically significant correlation between EDSS and PCS, i.e., EDSS and MCS values, in the group with the anxious and/or depressive reactive state (Table 4). 

## 4. Discussion 

In our research, all patients were without a confirmed diagnosis of anxiety or depression by a psychiatrist and they were not using any prescribed psychiatric therapy at the beginning of the rehabilitation. After testing by the HAD scale, the existence of an anxious or depressive reactive state was confirmed in 24 patients, while it was excluded in 34 patients. At the very beginning of processing the data, we observed that in patients without any reactive state, the period since making the diagnosis was 135.76 months, and in patients with reactive states, it was 69.50 months. There was a statistically significant difference in the duration of the disease in patients without anxious and/or depressive reactive states. At the same time, the average life age in the group without reactive states was 49.03 years, and in the group with reactive states, it was 49.88 years, i.e., there was no statistically significant difference, which excluded the impact of life age on the results of the research.

In our research, while monitoring the impact of rehabilitation on functionality, we compared the values of EDSS and BBS in both groups of patients at the beginning and at the end of the rehabilitation. The statistically highly significant difference in both groups was determined, i.e., there was a significant improvement of functionality, i.e., there was a decrease in EDSS values and an increase in BBS values. 

We also compared the values of these two parameters between group I and group II at the beginning and at the end of rehabilitation and determined that there was no statistically significant difference between the parameters of one versus the other group at the beginning nor at the end of the rehabilitation.

The next step was to examine the impact of rehabilitation on the QoL in patients with MS. The instrument used in our work for monitoring QoL of patients with MS through the rehabilitation process was SF36, i.e., we used two subscales—PCS and MCS. Both groups of patients had statistically highly significant differences after the rehabilitation compared to the beginning, in the sense of significant improvement in both their physical (PCS) and their mental condition (MCS). Thereafter, we compared the values of PCS and MCS between the groups at the beginning and at the end of rehabilitation. There was a statistically significant difference between the groups at the beginning versus at the end of rehabilitation in the PCS parameter, and there was a highly statistically significant difference in values of MCS. Patients in group I went into the rehabilitation process with a lower value of PCS compared to patients in group II, meaning that the patients of group I, according to their own evaluation of the physical condition, were worse compared to group II; although based on the objective assessment by a healthcare professional, there were no differences between the EDSS and BBS scales at the beginning of the rehabilitation.

The same ratio was observed at the end of the rehabilitation. Patients in group I did significantly improve their physical state, but to a much lesser degree compared to the patients in group II. A similar condition was observed with the values of the MCP subscale too. Although the patients of both groups had highly statistically significant improvements in MCS values, i.e., improvement of their mental conditions through the rehabilitation process, the patients of group II entered the treatment with a better mental state and, as such, were significantly better after the rehabilitation.

Considering that the QoL is mostly based on the subjective measurement of the subject’s perception of his or her own health condition, our final step was to determine if there was a correlation, in each group, between the EDSS parameter, measured by the health professionals, and the PCS and MCS, occurring as the patients’ subjective evaluation of their physical and mental condition. The analysis showed that there was a highly statistically significant correlation of the values of the EDSS and the PCS and the MCS in patients in group II, i.e., in the group where there was no reactive psychological state. Group I, which included patients with anxious and depressive reactive states, had no significant correlation in the objective evaluation by the health professionals, nor in the subjective evaluation by the patients.

We have observed that the psychologically reactive states of patients with MS significantly direct and shape the course and results of the rehabilitation process, regardless of treatments that have been shown to be effective. The studies [30] have shown that the level of anxiety in patients with newly diagnosed MS and their partners is high. These findings support the fact that anxiety can be a reactive psychological reaction to the disease, and not an independent disease nor a consequence of lesions that occur on the brain [14].

Research on the possible connection between MS and depression has shown that changes in certain parts of the brain during MS were linked to the onset of depression [31,32,33]. However, theories about the etiology of depression are still not unanimous. There are indications that depression, just like anxiety, could be a reaction to the unpredictable nature of the underlying disease and the disability that is arising as a result [34]. 

The literature data indicate that anxiety often occurs in newly diagnosed patients and the threatening morbidity with depression increases the rate of suicidal ideas [14]. The occurrence of psychological symptoms can be seen in patients with MS even before making the diagnosis when the patients sense the first unexpected neurological symptoms, leading to the feeling of vulnerability [35,36]. Patients have reported conflicts in emotional reactions, such as shock, anxiety, fear, sadness, anger, insecurity, shame, loss of identity, abandonment, and loss of self-confidence, which plays a role in the emergence of psychiatric changes. Some patients have even expressed the appearance of a sense of relief after finding out the diagnosis, which directed them to look for help, hope, and acceptance [35]. 

Our research showed us that patients with a longer duration of the disease were more adapted to it, with already emotionally and cognitively processed information, and were aligned with the environment, family, social, and professional requirements and activities regardless of the severity of the disease, even though the severity of the disease, in some patients without reactive states, was up to 8, i.e., 8.5, according to EDSS. We know that MS is a disease that most often gets diagnosed at a younger life age, and this difference in the duration of the disease between the groups can be related to the fact that the patients with confirmed disease of such progressive character, without a possibility of a cure, need time to come to terms with such emotional shock [37] and to cope with the future challenges. It is no less challenging for patients to adapt to life with the disease, to accept it without denying it, and to provide themselves with a quality life. 

Physical activity is very important to patients with MS from the very beginning of the disease. The real result is obtained by an organized physical activity through the rehabilitation process. Supervised therapy is important because of the individualized assessment, the follow-up, and the adjustment of interventions [11]. 

Physiotherapy, as an integral part of rehabilitation with its kinesiotherapeutic action, has a goal of improving the patient’s mobility through compensatory mechanisms that include the activation of effectors and restoring the function with active mental commitment, and is not an isolated movement [12]. The degree of activities depends on the patient individually, and fatigue and overheating of the body must be monitored, making sure that the spasticity is not increased [38]. 

The basis of the treatment in our work was the application of the Bobath concept. It represents a method that restores natural human movement [39]. The assumption is that the essence of motoric deficits occurring due to diseases of CNS is in postural reflexes needed for coordination in space. Correct muscle tone and active movements can be gained by inhibiting pathological postural patterns [40]. The advantage of the concept in everyday work is the possibility to position the patient in all positions, which enables working with patients with severe motor deficits. Disturbance of balance and equilibrium in patients with MS has been seen regularly, and we also carried out exercises for restoration of these functions in our research. With this, we improved posture control, from the balance in the seated position to increased stability when standing and walking. EDSS is an assessment instrument and represents the gold standard in everyday practice for patients with MS [22]. The second parameter that was used to monitor the success of the physical therapy was BBS. Persons with MS are at a higher risk of falls than the general population and older subjects are, with a reported fall prevalence ranging from 48% to 63% [41]. It has been determined [42] that 63.5% to 82.6% of patients report fear of falling, thus reducing their activities. These patients fall with a higher possibility of injury, often indoors, even for younger individuals with minor disabilities. 

Our results showed the importance of rehabilitation in patients with MS, which was confirmed by basic brain research. Brain MRI showed that after eight weeks of kinesiotherapy, there was an improved connection between the regions of the brain in patients with mild sensorimotor deficits of the upper extremities and restoration of brain activity which coincided with a reduction of compensatory activities of other regions of the brain and reduced brain damage [43].

As a chronic disease, MS has a significant effect on the life of patients. QoL of patients with MS is not systematically evaluated in a routine clinical praxis. QoL is mostly based on a subjective measurement of the subject’s perception of his or her own health condition through standardized tools and instruments offering a quantitative method for monitoring an individual’s health condition [37]. The patient’s perception of their own QoL may even predict the future progression of the disease and disability [44,45].

Previous studies have already shown that patients and doctors do not agree on which health domains are the most important in MS [45]. These results point us to the fact of how significant the psychological reaction to the disease is in MS patients, as our results indicated, too. Health professionals gain insight into patients’ improvement according to standardized test assessments, but the patients’ real experience of their health conditions, which is often crucial in the recovery, has a different sign. This confirms the need to include the QoL tests in the everyday assessment of the patient’s condition, the progression of the disease, and the success of the therapy, which we also recommend.

The strength of our study was that all data were prospectively collected for the first time in our country, with the exposure and outcomes collated independently, thereby effectively eliminating the potential for recall and related biases. The limitation of the study was its small sample of patients, which left us unable to divide them according to the degree of disability. Also, there is a need to include psychiatrists in patient evaluations prior to rehabilitation. In perspective, control exams and rehabilitation once a year should give us the information on long-term evaluation of functionality and QoL of these patients. 

## 5. Conclusions

Although rehabilitation leads to an objective improvement of functionality in patients with MS, the presence of the anxious and/or depressive reactive state limits the results of rehabilitation and leads to discrepancies in the aforementioned objective assessment and in the patient’s subjective experience through the evaluation of QoL.

## Figures and Tables

**Table 1:** Descriptive statistics in both groups.

	Group I		Group II		*p*
	Mean	SD	Mean	SD	
**Age (years)**	49.88	7.652	49.03	11.376	0.752
**Beginning of the disease (In months)**	69.50	60.543	135.76	102.658	0.006 *
**EDSS before therapy**	5.23	1.567	5.38	1.954	0.751
**EDSS after therapy**	4.40	1.482	4.82	1.766	0.337
**PCS before therapy**	32.08	14.870	44.50	15.258	0.003 *
**PCS after therapy**	41.13	15.607	53.18	17.827	0.01 *
**MCS before therapy**	37.54	17.922	56.15	14.565	0.000 **
**MCS after therapy**	47.08	18.960	65.18	16.941	0.000 **
**BBS before therapy**	25.25	10.711	23.65	11.708	0.597
**BBS after therapy**	33.29	11.442	31.65	12.780	0.616

EDSS ranges between zero and 10; PCS and MCS range from zero to 100; BBS ranges from zero to 56; * statistically significant difference; ** high statistical significance.

**Table 2:** Comparison of values of BBS, EDSS, PCS, and MCS before and after the rehabilitation in both groups.

		Group I			Group II		
		Mean ± SD	*t*	*p*	Mean ± SD	*t*	*p*
**BBS—before**	**BBS—after**	−8.042 ± 6.537	−6.026	0.000	−8.000 ± 8.904	−5.239	0.000
**EDSS—before**	**EDSS—after**	0.833 ± 0.654	6.244	0.000	0.559 ± 0.587	5.548	0.000
**PCS—before**	**PCS—after**	−9.042 ± 14.076	−3.147	0.005	−8.676 ± 11.211	−4.513	0.000
**MCS—before**	**MCS—after**	−9.542 ± 14.377	−3.251	0.004	−9.029 ± 14.494	−3.632	0.001

**Table 3:** BBS, EDSS, PCS, and MCS between groups before and after rehabilitation.

	Levene’s Test for Equality of Variances		*t*-Test for Equality of Means						
	F	Sig.	t	df	Sig. (2-Tailed)	Mean Difference	Std. Error Difference	95% Confidence Interval of the Difference	
								Lower	Upper
**EDSS—before**	2.799	0.100	−0.318	56	0.751	−0.153	0.481	−1.117	0.811
**EDSS—after**	2.490	0.120	−0.969	56	0.337	−0.428	0.441	−1.312	0.456
**BBS—before**	0.249	0.619	0.532	56	0.597	1.603	3.015	−4.437	7.643
**BBS—after**	1.579	0.214	0.504	56	0.616	1.645	3.265	−4.897	8.186
**PCS—before**	0.182	0.671	−3.084	56	0.003 **	−12.417	4.026	−20.481	−4.352
**PCS—after**	0.382	0.539	−2.667	56	0.010 *	−12.051	4.519	−21.104	−2.999
**MCS—before**	0.979	0.327	−4.354	56	0.000 **	−18.605	4.273	−27.166	−10.045
**MCS—after**	0.494	0.485	−3.813	56	0.000 **	−18.093	4.745	−27.599	−8.588

* statistically significant difference ** highly significant difference.

**Table 4:** Correlation of EDSS with PCS and MCS at the end of rehabilitation in both groups.

		Group I		Group II	
		PCS	MCS	PCS	MCS
**EDSS**	**r**	0.171	−0.498 **	−0.498 **	−0.437 **
	**p**	0.425	0.003	0.003	0.010
**BBS**	**r**	−0.271	0.166	0.166	0.182
	**p**	0.199	0.347	0.347	0.304

** highly significant difference.

## Data Availability

Data are contained within the article.
